# A study on the changes in the microstructure of trabecular bone and the biomechanical properties in patients with osteoporotic fractures and the correlation between them

**DOI:** 10.3389/fmed.2026.1932467

**Published:** 2026-10-16

**Authors:** Hao Liu, Xu Zhang, Wenjie Xu, Pei Yang, Zhizheng Xiong

**Affiliations:** 1 Department of Orthopedic, Yueyang Hospital Affiliated to Hunan Normal University, Yueyang, Hunan, China; 2 Department of Orthopedic, Yueyang People’s Hospital, Yueyang, Hunan, China; 3 Department of Orthopedic, Second Affiliated Hospital of Xi'an Jiaotong University, Xi'an, Shanxi, China

**Keywords:** biomechanics experiments, bone microstructure, femoral neck fracture, osteoporosis, trabecular bone

## Abstract

**Objective:**

To explore bone-quality characteristics in patients with osteoporotic femoral neck fractures by investigating trabecular-bone microstructural and biomechanical alterations of femoral-head specimens and their correlations.

**Methods:**

Femoral-head specimens from 20 patients with osteoporotic femoral neck fractures (experimental group: 69-90 years, 80.65 ± 8.85 years) and 13 patients with early-stage hip degenerative changes (control group: 49-83 years, 64.08 ± 6.93 years) were included. Noticeable baseline age imbalance existed between groups. All specimens underwent Micro-CT scanning. Cylindrical 8-mm-diameter trabecular-bone samples were harvested along principal compressive trabeculae. Compression, bending and torsion tests were conducted to acquire microstructural and biomechanical parameters.

**Results:**

Compared with controls, the experimental group exhibited significantly lower bone volume fraction (BV/TV), trabecular number (Tb.N), trabecular thickness (Tb.Th), alongside higher trabecular separation (Tb.Sp) and structural model index (SMI) (all *P*< 0.05). Biomechanical indicators including maximum compressive stress (MCL), maximum bending force (Fmax) and maximum torque (RST) were markedly reduced in the experimental group (*P* < 0.05). Trabecular microstructural indices were significantly correlated with biomechanical performance. Of note, baseline age imbalance and potential confounding from hip degenerative pathology should be considered when interpreting inter-group differences.

**Conclusion:**

This ex-vivo specimen-based study demonstrates severe trabecular deterioration and impaired biomechanical properties in osteoporotic-fracture specimens, with significant correlations between microstructural damage and reduced mechanical performance. These observations only reflect bone-quality features of isolated specimens; direct extrapolation to predict individual *in-vivo* fracture risk should be avoided.

## 1 Introduction

Osteoporosis (OP) is a systemic skeletal disease characterized by a decrease in bone mass, deterioration of bone microarchitecture, increased bone fragility, and a higher susceptibility to fractures (1). Osteoporosis in clinical practice often leads to spinal deformities, fractures in multiple sites such as vertebrae and hips, resulting in disability and death, and is referred to as a silent killer. With the acceleration of the aging process in our country, osteoporosis poses a serious threat to the health of the middle-aged and elderly population. According to the latest large-sample epidemiological survey, the age-standardized prevalence rates of osteoporosis among individuals over 50 in our country are 6.46% for males and 29.13% for females (2).

The current gold standard for diagnosing osteoporosis is bone density testing, which can only reflect changes in bone mass, and its specificity and sensitivity in predicting fracture risk are relatively low (3). The mechanical strength of bone tissue is not only related to the mineral density of the bone but is also closely associated with the microstructure of trabecular bone. In recent years, there has been an increasing amount of research on the microstructure of trabecular bone in studies related to osteoporosis (4). Therefore, changes in bone microstructure have a significant impact on the occurrence of osteoporosis.

The microstructural characteristics of bone are an important determinant of bone strength, bone quality, and mechanical properties, yet they are quite challenging to assess in the human body (5). With the improvement of basic research levels, there have been many advanced methods for studying bone quality. In recent years, the application of *μ*CT, fatigue testing machines, and mechanical testing machines to study the microstructure and mechanical properties of trabecular bone represents a new technology and approach that can intuitively reflect changes in bone quality. Osteoporotic fractures primarily occur in areas rich in cancellous bone—such as the hip, thoracolumbar vertebrae, distal radius, proximal humerus, and ankle—among which hip fractures are the most severe (6). In this study, we intend to explore the correlation between bone microstructure and biomechanics through observation and experimentation, which can provide theoretical support for the pathogenesis, early diagnosis, and treatment of bone diseases.

## 2 Materials and methods

### 2.1 Research object

The specimens of femoral heads after joint replacement surgery were collected from patients with osteoporotic femoral neck fractures (fracture group) and patients with degenerative changes of the hip joint (control group) who were hospitalized in the Department of Orthopedics at the Yueyang People’s Hospital. The fracture group met the diagnostic criteria for fragility fractures (low-energy trauma: falls from standing height or <1 meter). The control group comprised patients with early degenerative changes (cartilage-initiated pathology without advanced bone loss or trabecular structural damage). By analyzing medical history, imaging examinations, and pathological examinations, femoral heads from patients undergoing hip joint replacement surgery with bone tumors, rheumatoid arthritis, and ankylosing spondylitis, and femoral head necrosis were excluded. Rationale for control group selection: Control specimens were obtained from identical orthopedic joint-replacement surgical setting. Specimen harvesting, preservation, Micro-CT scanning and biomechanical testing adopted unified experimental workflows for both groups. Fresh normal human femoral head specimens are ethically unavailable for this ex-vivo study. Among them, there were 20 cases of femoral neck fracture patients (ages 69 to 90 years [(80.65 ± 8.85) years]) and 13 cases of femoral heads from patients with degenerative changes of the hip joint (ages 49 to 83 years [(64.08 ± 6.93) years]). All patients had complete clinical relevant case data, including current medical history and past medical history, to ensure matching in basic aspects such as gender. Noticeable age difference existed between two groups; age-matching was not achieved in specimen recruitment.

### 2.2 Specimen preparation

The specimens will be preserved in 10% formalin neutral buffered solution. After completing the micro-CT scan of the entire femoral head specimen, the femoral head will be positioned as *in vivo*, followed by localization marking. The femoral neck specimen will be divided into three equal parts from front to back, and the middle section will be extracted using a hollow saw (*φ*8 mm) to obtain an 8 mm diameter cylindrical piece of cancellous bone within the maximum diameter perpendicular to the stress direction of the femoral head (see Figure 1), which will then be preserved in 10% formalin neutral buffered solution. All specimens in both groups adopted identical 10% neutral buffered formalin fixation conditions. Cylindrical trabecular bone specimens (8 mm diameter) for compression, bending and torsion mechanical tests were all harvested from the maximal-diameter zone of the femoral head along the orientation of principal compressive trabeculae. To minimise sampling variability, sampling location and orientation were marked on each intact femoral head before cutting; only the middle slice after three-part front-to-back segmentation of femoral neck was sampled, and all sampling operations were completed by one operator following a unified standard operating procedure.

**Figure 1:**
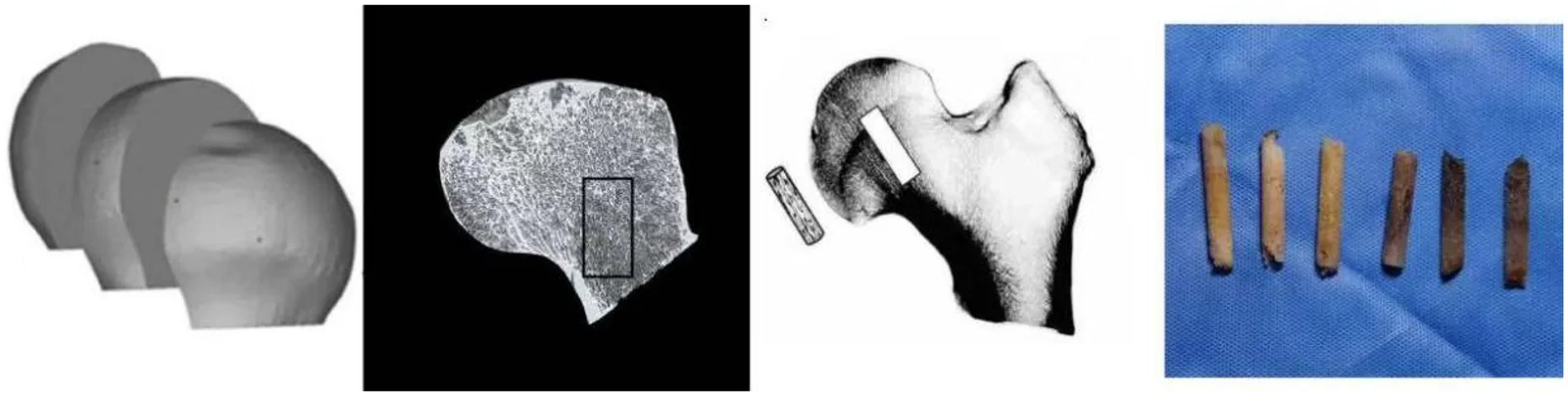
Schematic diagram of the selected region of cancellous bone in the femoral neck area.

### 2.3 Micro-CT scan

After fixing the sampled specimens, Micro-CT scanning was performed (model: SkyScan 1276, Bruker, Germany) (Figure 2). The scanning parameters were: tube current of 200uA, voltage of 100KV, scanning the entire object, scanning resolution of 36.000480μm, exposure time of 211 ms, and scanning angle of 360 degrees. Calibration was conducted using a phantom scanned under the same conditions (provided by the equipment manufacturer). The original images were obtained after scanning. The original images were reconstructed in the selected area using the three-dimensional reconstruction software NRecon (software version V1.7.4.2, Bruker, Germany). Before reconstruction, a preview of the reconstructed images was performed. To reduce image artifacts and achieve optimal reconstruction results, the reconstruction parameters were adjusted to the following values: Smoothing=5, Beam-hardening=8, Ring artifacts=25%, and other parameters. Once the parameters were set, the specified folder was edited to begin image reconstruction. The CT Analyser (software version 1.20.3.0, Bruker, Germany) was used to analyze the region of interest (ROI). Unified parameters were set, and the software calculated the total volume (TV), bone volume (BV), volume ratio (BV/TV), bone area (BS), number of trabecular bones (Tb. N), and trabecular thickness (Tb.th), among other parameters. Identical reconstruction parameters and region-of-interest threshold settings were applied for all specimens to reduce inter-sample systematic measurement bias. Partial-volume artefacts cannot be completely eliminated under current 36 μm scanning resolution.

**Figure 2:**
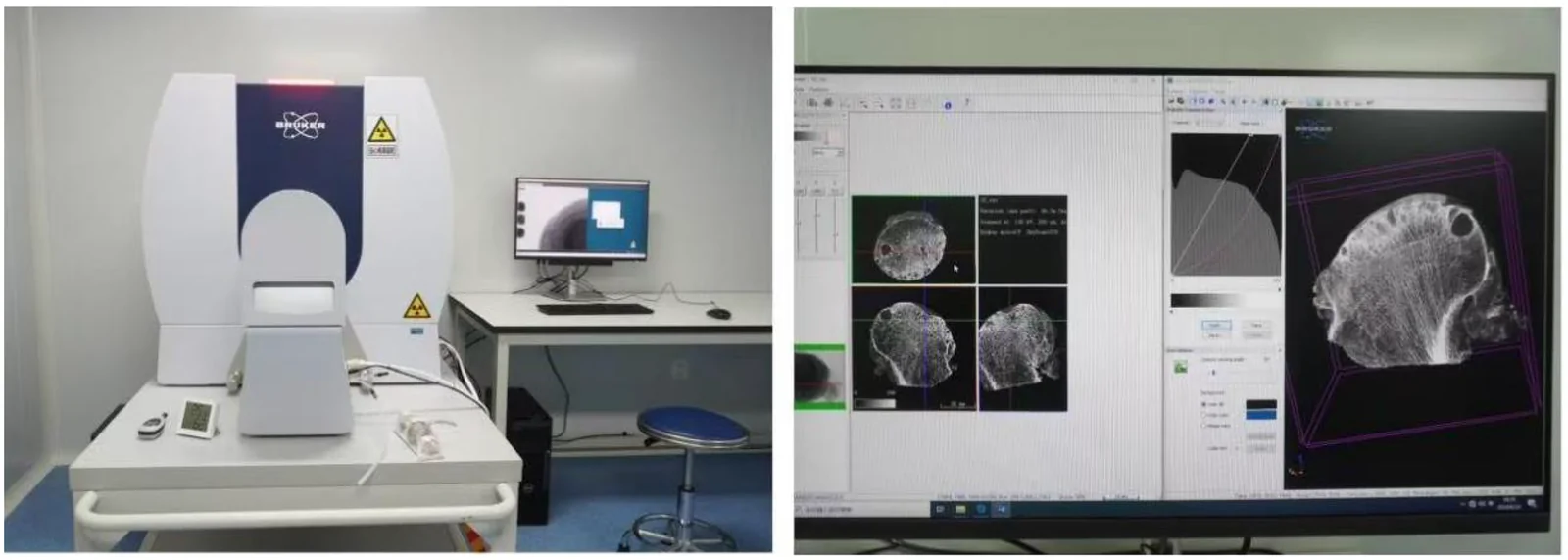
Micro-CT scan.

### 2.4 Biomechanics experiment

#### 2.4.1 Compression experiment

The specimen was removed from the 10% formalin neutral tissue fixative and placed vertically with an approximate diameter of 3 mm on the platform of the electro-hydraulic servo fatigue testing machine (Hualong PWS-10, Shanghai Hualong Testing Instrument Co., Ltd., China) (Figure 3). The basic parameters in the computer operating software are as follows: the beam descent speed is 25 mm/min, the compression degree is 10%, and the initial condition for the experiment is a force of 10N. The condition for the end of the experiment is the appearance of a peak force in the compression curve, with a significant downward trend in the curve. The load-deformation curve was obtained, and the maximum force when the specimen was compressed by 10% was recorded, with the highest point of the curve representing the maximum load (Figure 4).

**Figure 3:**
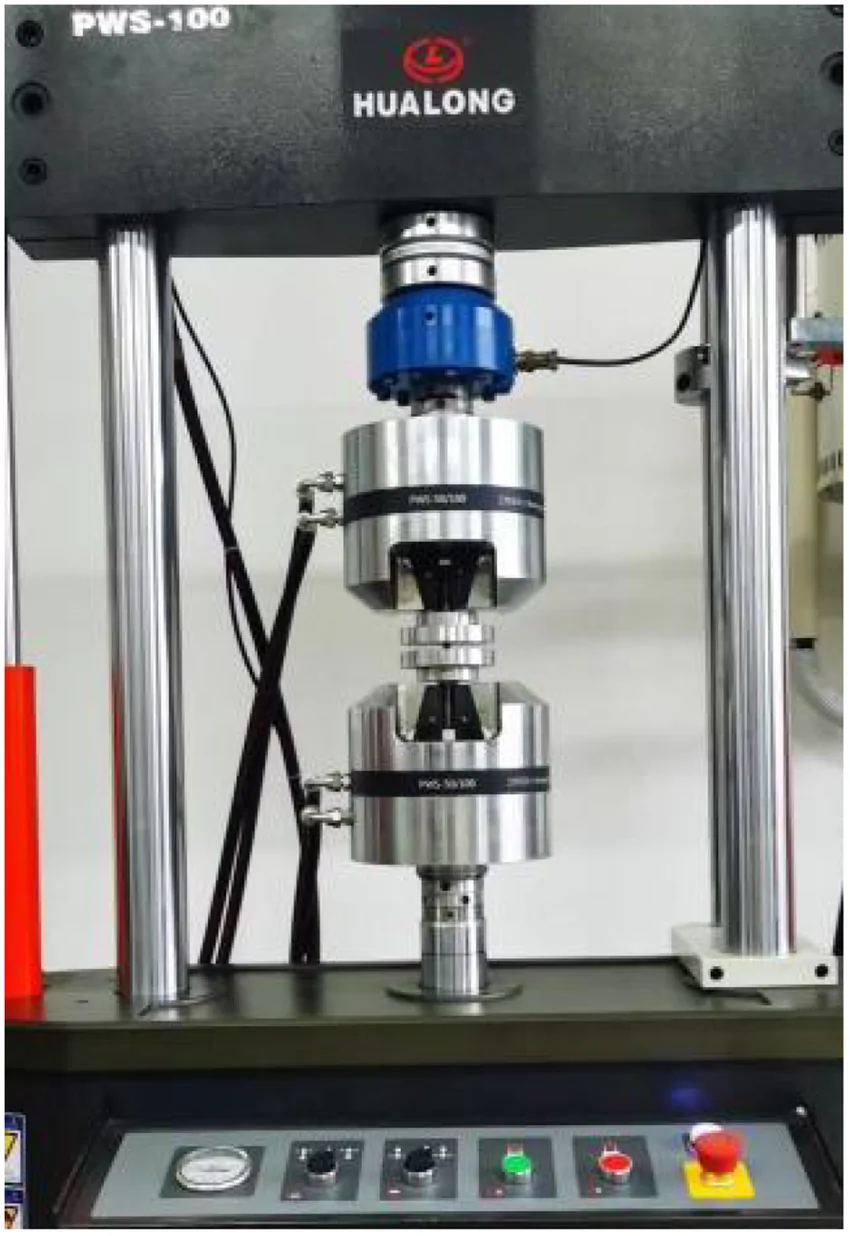
Electro-hydraulic servo fatigue testing machine.

**Figure 4:**
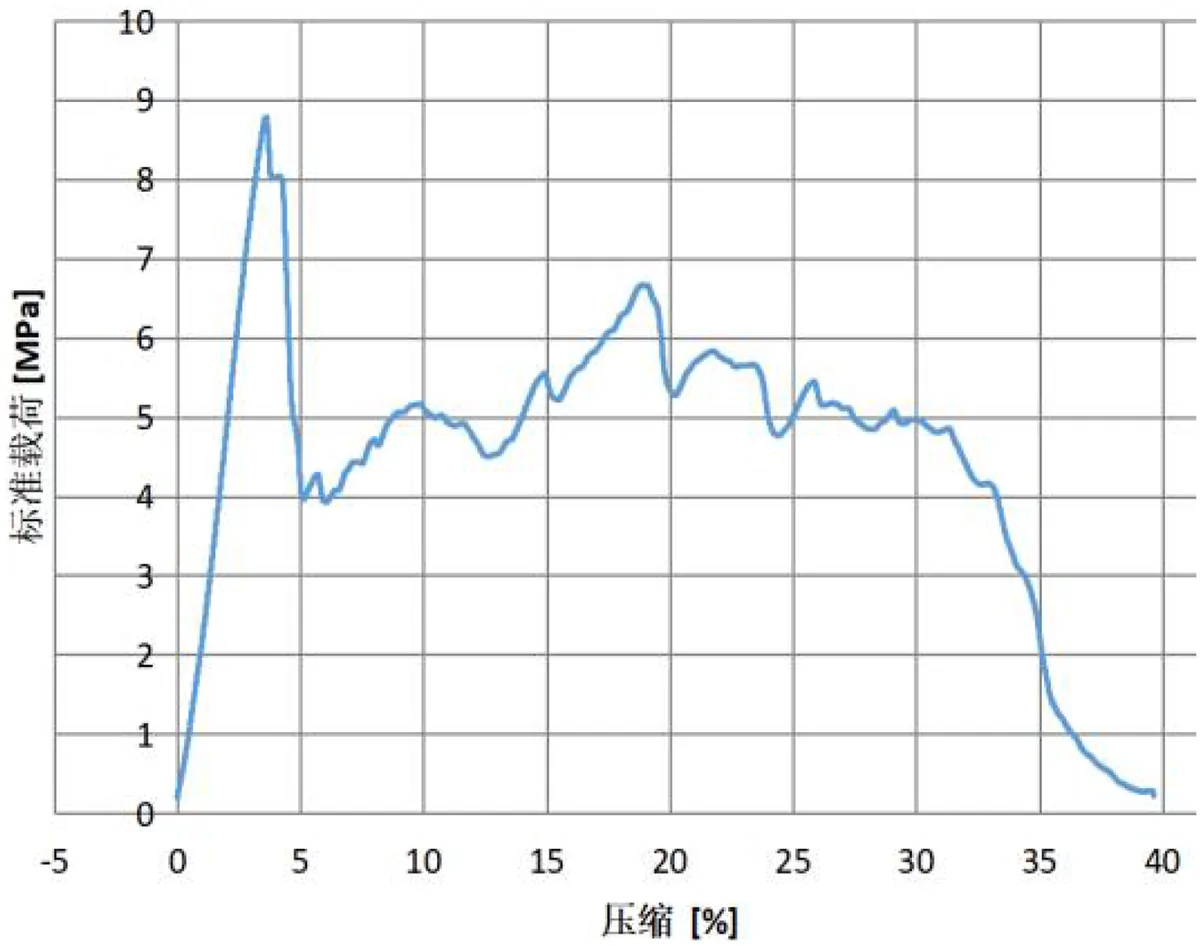
Load-deformation curve.

#### 2.4.2 Flexural test

Specimens were removed from 10% neutral-buffered formalin and visually inspected to exclude surface defects. Each specimen was mounted on a three-point bending fixture of an electro-hydraulic servo fatigue testing machine (Model PWS-10, Hualong Test Instruments Co., Ltd., Shanghai, China) (Figure 5) with a 20 mm span, ensuring alignment between the specimen central axis and loading head. Testing parameters included a loading rate of 25 mm/min. The machine was initiated, and the load-displacement curve was monitored in real-time. The test was terminated immediately upon audible or visual fracture indication (defined as >10% load drop from peak force), with the maximum load at fracture recorded as flexural strength.

**Figure 5:**
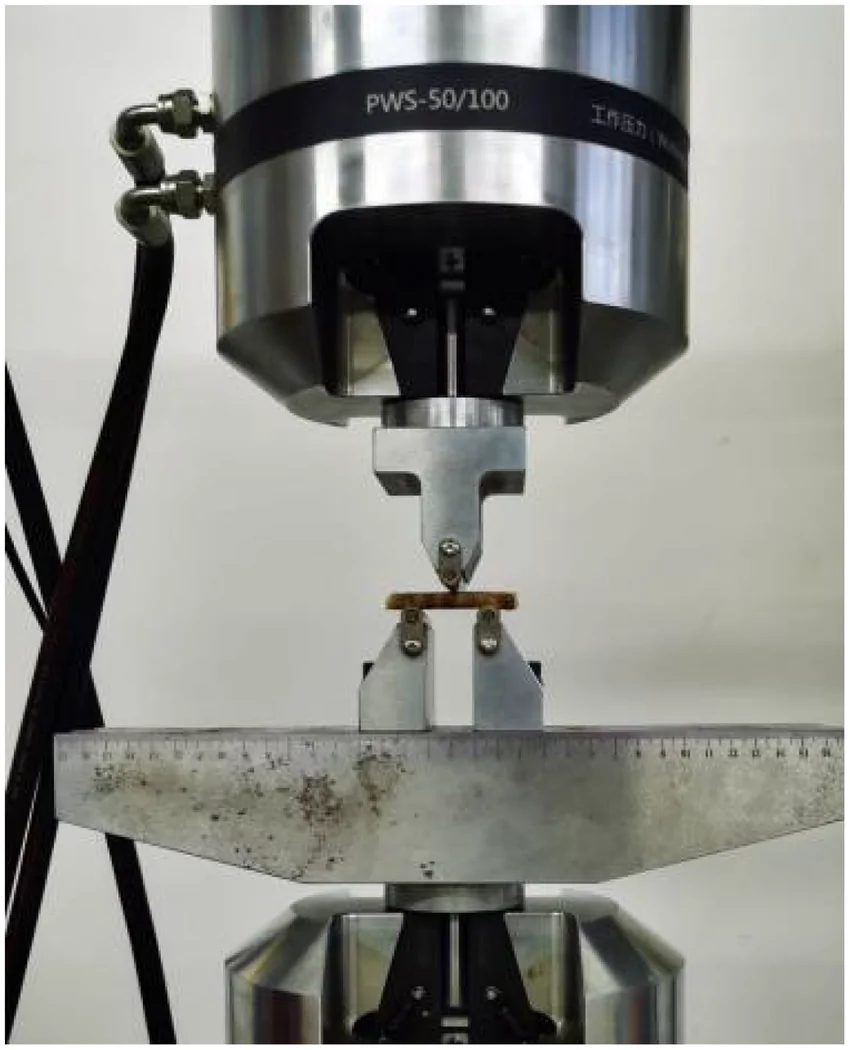
Folding resistance experiment.

#### 2.4.3 Anti-torsion test

Remove the specimens from the 10% formalin neutral tissue fixative, ensuring that all specimens are of uniform length. Fix both ends in a specific mold (Figure 6), place the experimental specimens on the interface shear instrument (KTL-IST, Xi'an Kangtuoli Instrument Equipment Co., Ltd., China) (Figure 7), and set the basic parameters in the computer operating software as follows: set the rotation speed to 30°/min. Start the testing machine to load the bone specimens at the specified rotation speed. End of experiment conditions: a peak force appears in the curve, and the curve shows a significant downward trend, recording the maximum torque of the specimen, that is, the highest point value of the curve is the maximum torque.

**Figure 6:**
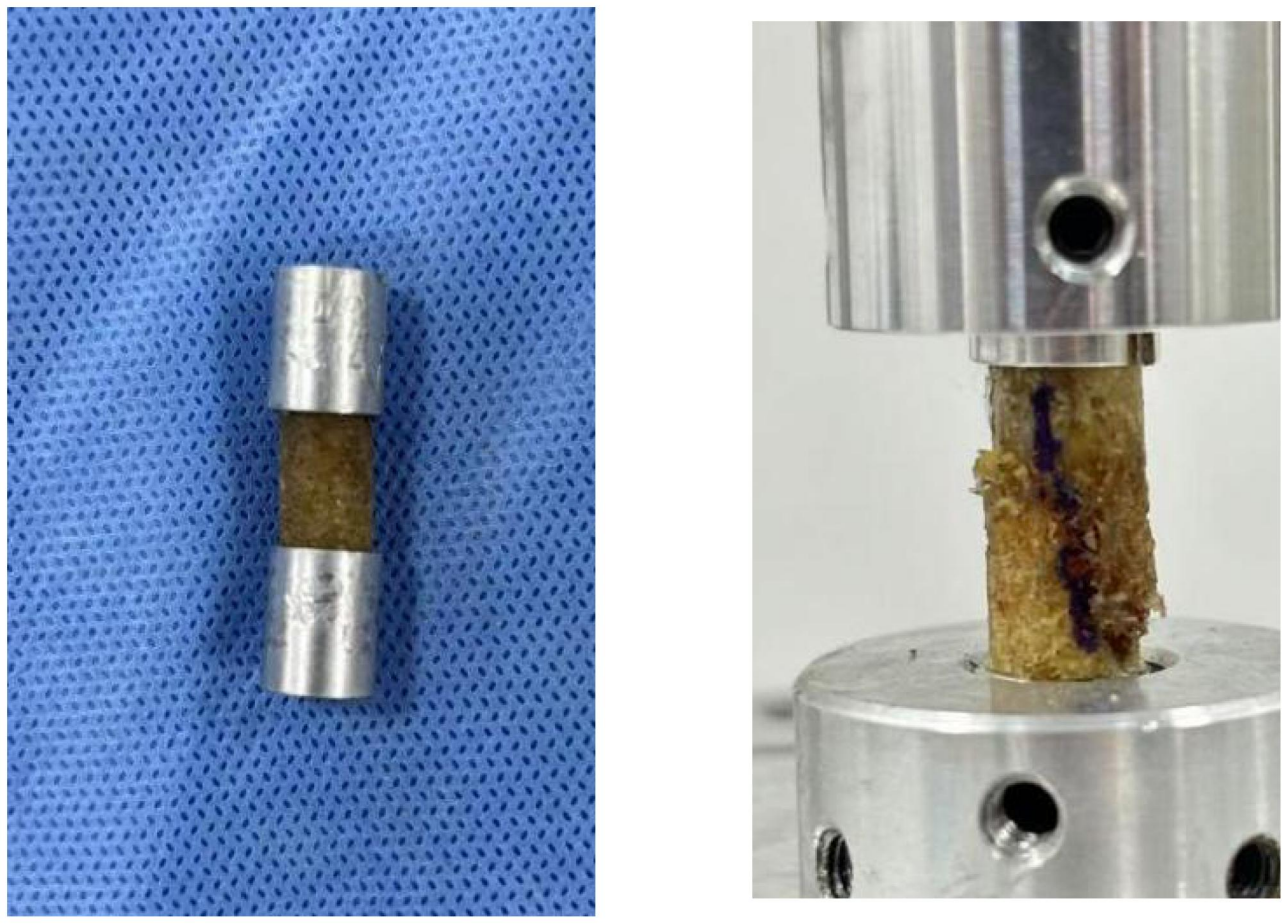
Specimen fixation mould.

**Figure 7:**
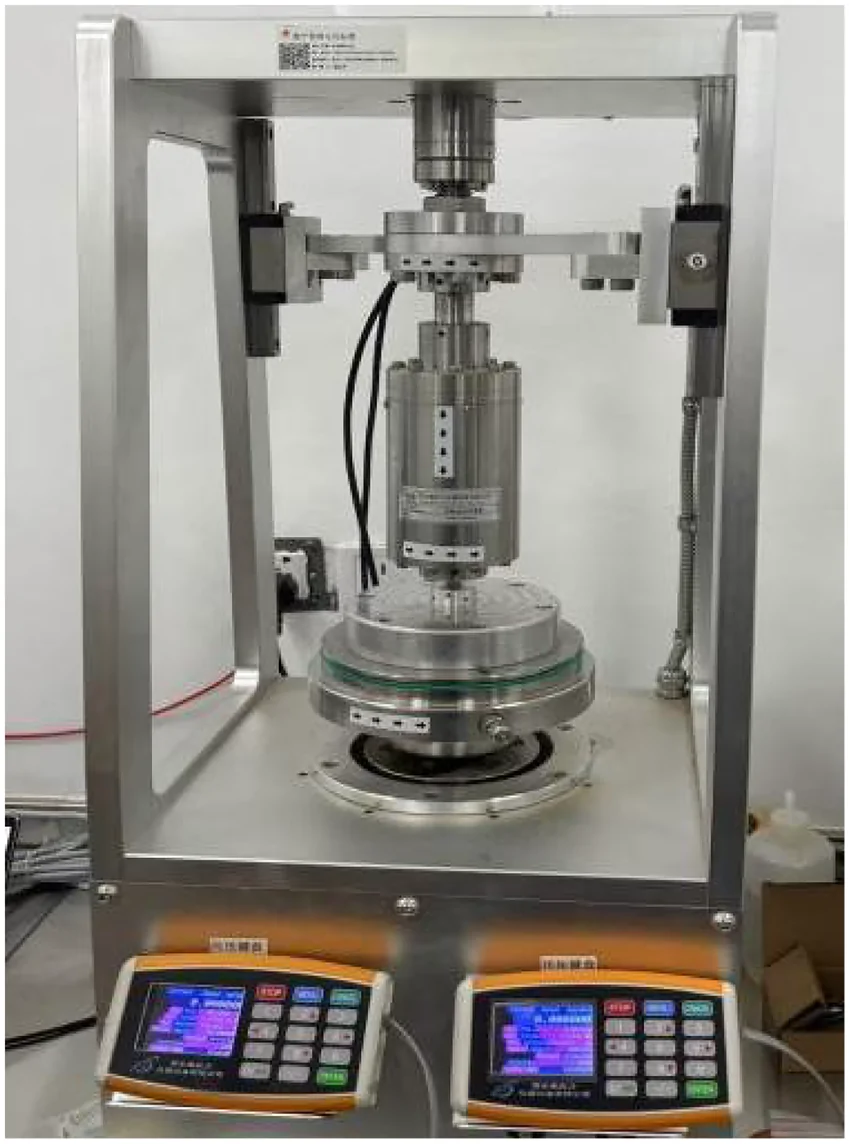
Interface shear.

#### 2.4.4 Statistical methods

All statistical analyses were conducted using the SPSS 27.0 (IBM SPSS Statistics 27 for Mac) software package, and all graphics were created using the SPSS 27.0 (IBM SPSS Statistics 27 for Mac) software package and Prism 9.5 (GraphPad Prism 9.5) software package. Normality tests and homogeneity of variance tests were performed on the two groups of data. For samples with non-strict normal distribution and unequal variances, the results were expressed as medians and interquartile ranges. The differences in medians between groups were compared using the non-parametric Mann–Whitney test, and correlation analysis was conducted using Spearman correlation analysis, with *p* < 0.05 considered statistically significant. Of note, obvious age imbalance existed between two groups. Age matching was not performed during specimen recruitment, and age was not included as covariate for statistical adjustment in current analysis. Of note, obvious age imbalance existed between two groups. Age matching was not performed during specimen recruitment, and age was not included as covariate for statistical adjustment in current analysis. Multiple-comparison correction was not conducted for Spearman correlation analyses; correlation results should be regarded as exploratory findings.

## 3 Result

### 3.1 Comparison of the microstructure and biomechanical parameters of two groups of cancellous bone

Compared to the control group, the trabecular thickness (Tb.Th), trabecular number (Tb.N), and bone volume fraction (BV/TV) of the femoral head in the fracture group decreased, showing statistical significance (*P* < 0.05). The trabecular separation (Tb.Sp) and structural model index (SMI) increased, also with statistical significance (*P* < 0.05). The remaining structural indicators showed no statistical significance; compared to the control group, the maximum torque (RST), maximum compressive stress (MCL), and maximum bending strength (Fmax) of the femoral head in the experimental group decreased, all showing statistical significance (*P* < 0.05) (see Table 1; Figures 8, 9).

**Table 1:** Comparison of microstructural and biomechanical parameters between the two data samples.

Parameters	Experimental group (*n* = 20)	Control group (*n* = 13)	Z	*P*
BV/TV (%)	26.98 (23.39−30.44)	36.99 (30.28–46.54)	−3.021	0.
SMI	0.90 (0.64∼1.07)	0.09 (−0.62 to 0.92)	−2.358	0.
Tb.Th(mm)	0.30 (0.28∼0.32)	0.45 (0.34 to 0.52)	−3.371	0.
Tb.N(1/mm)	0.80 (0.75∼0.85)	0.93 (0.86∼0.99)	−3.390	0.
Tb.Sp(mm)	0.92 (0.87∼0.96)	0.82 (0.78∼0.87)	−2.542	0.
MCL(MPa)	4.38 (3.25–5.12)	8.79 (8.15–9.43)	−4.606	0.000**
Fmax(N)	105.99 (94.82–132.77)	254.49 (217.20–283.32)	−4.458	0.000**
RST (Nm)	0.32 (0.26–0.40)	0.79 (0.72–0.95)	−4.496	0.000**

* *p* < 0.05 statistically different, ** *p* < 0.01 statistically different.

**Figure 8:**
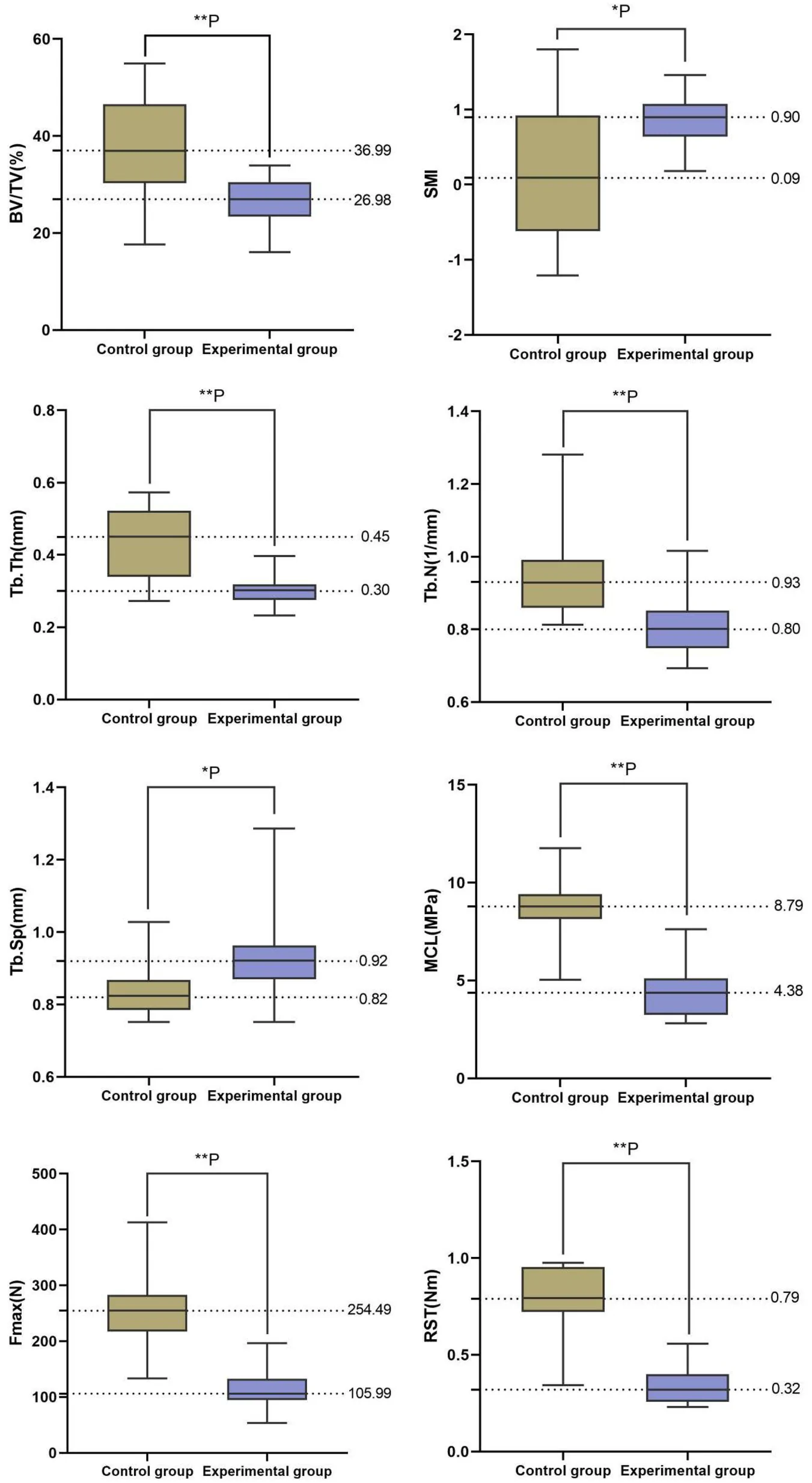
Comparison of median and lower upper quartile of microstructural and biomechanical parameters of the two data samples.

**Figure 9:**
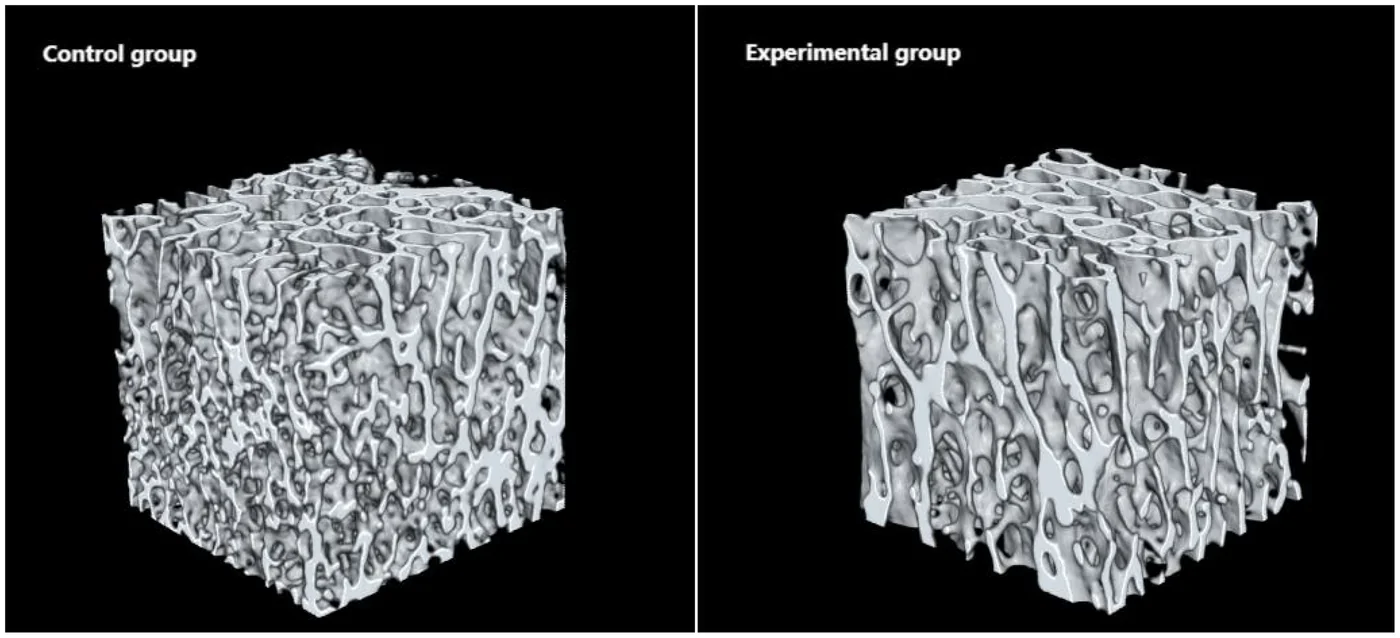
Comparison of three-dimensional visualization imaging of trabecular bone between the experimental group and the control group.

### 3.2 The correlation between the microstructure of trabecular bone and biomechanical parameters

The application of Spearman correlation analysis indicates that the parameters of trabecular microstructure, Tb.N, Tb.Th, and BV/TV, are positively correlated with the MCL, RST, and Fmax of the femoral head cancellous bone (*P* < 0.05), while Tb.Sp and SMI are negatively correlated with the MCL, RST, and Fmax of the femoral head cancellous bone (*P* < 0.05). (See Table 2, Figure 10).

**Table 2:** Correlation between the microstructure of two groups of data samples and biomechanical parameters.

Parameter	MCL(MPa)		Fmax(N)		RST(Nm)	
	*r*	*p*	*r*	*p*	*r*	*p*
BV/TV(%)	0.562	0.001**	0.555	0.001**	0.710	0.000**
SMI	−0.523	0.002**	−0.524	0.002**	−0.599	0.000**
Tb.Th(mm)	0.539	0.001**	0.515	0.002**	0.706	0.000**
Tb.N(1/mm)	0.496	0.003**	0.432	0.012*	0.701	0.000**
Tb.Sp(mm)	−0.400	0.021*	−0.544	0.001**	−0.452	0.008**

* *p* < 0.05 statistically different, ** *p* < 0.01 statistically different.

**Figure 10:**
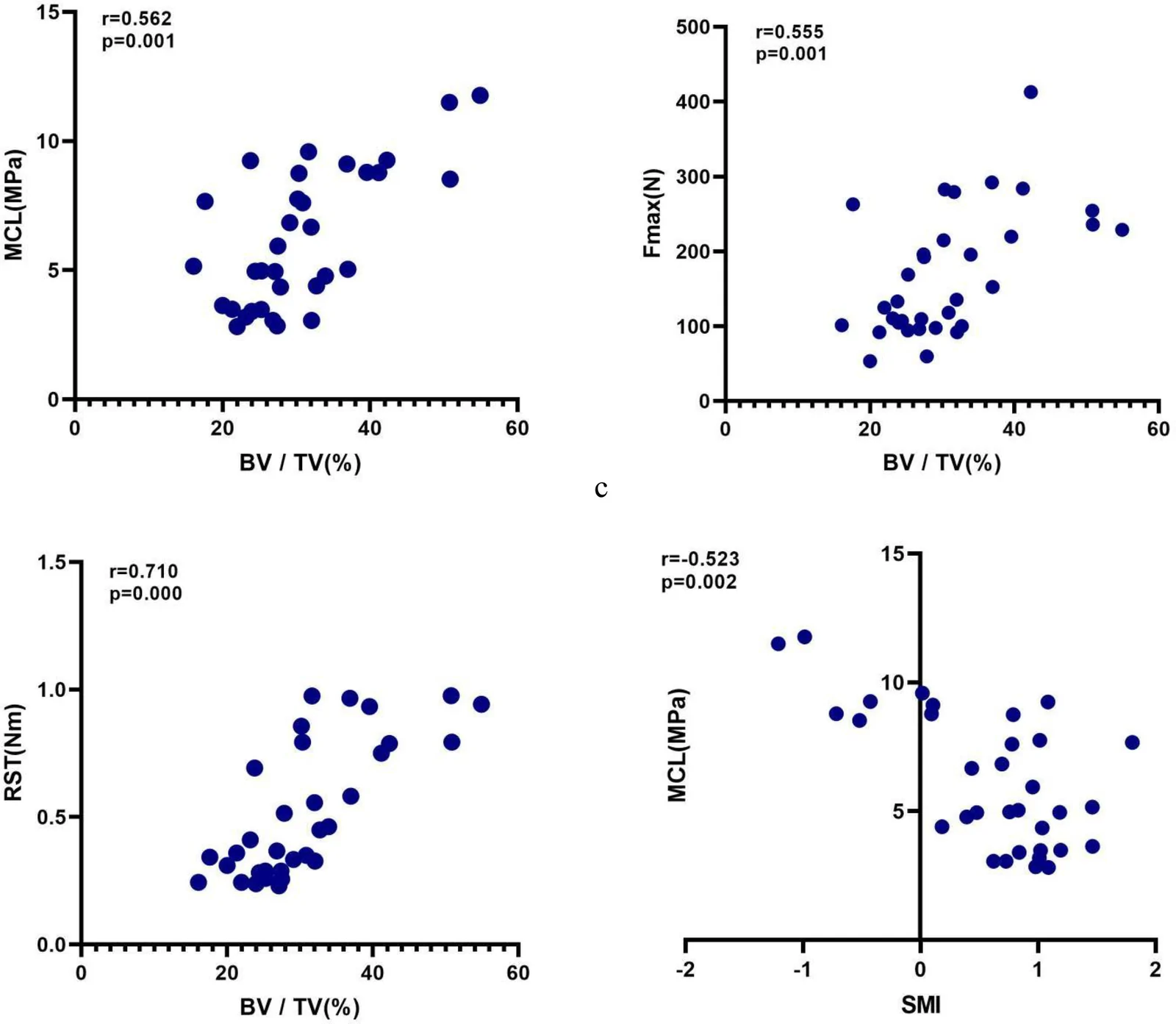

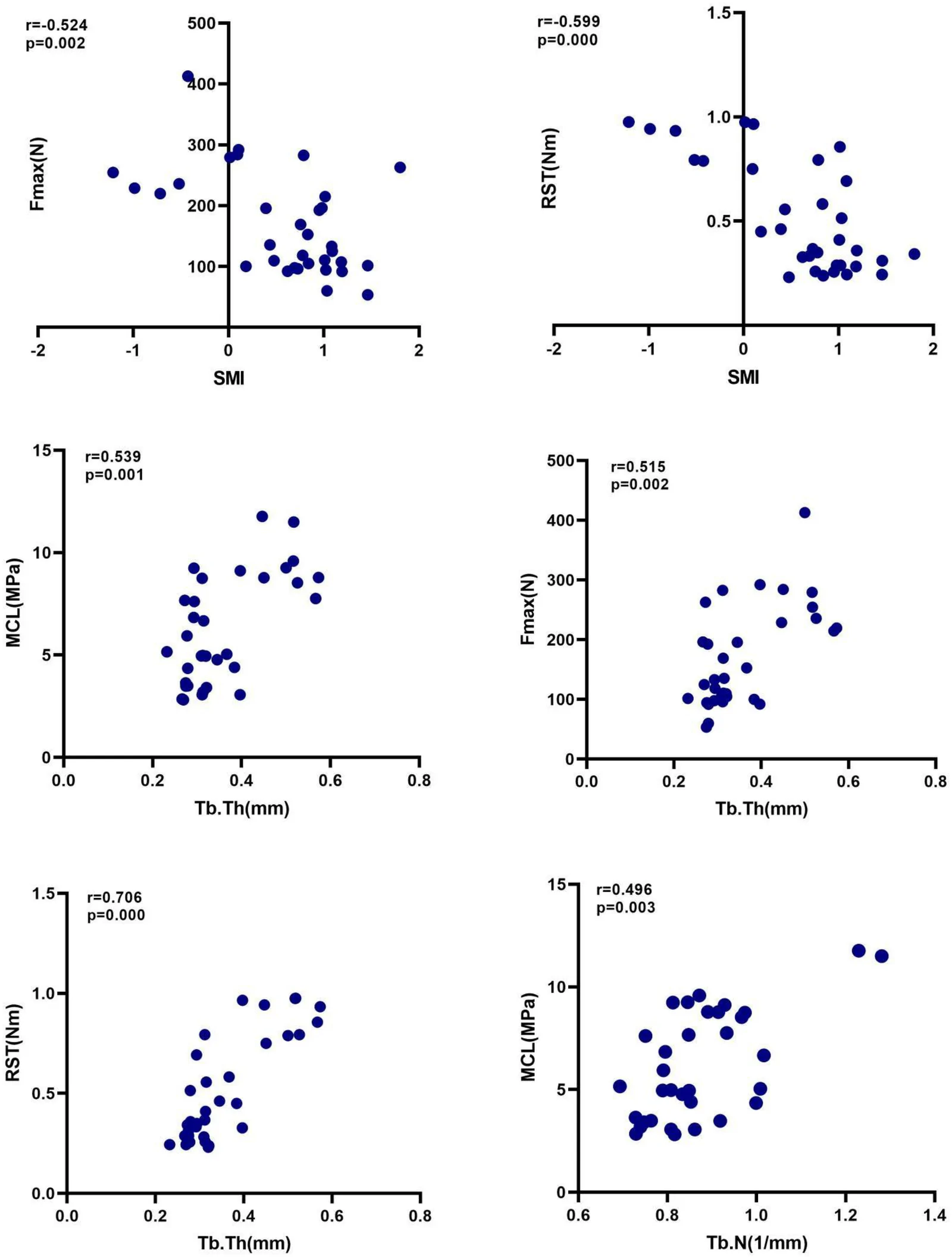

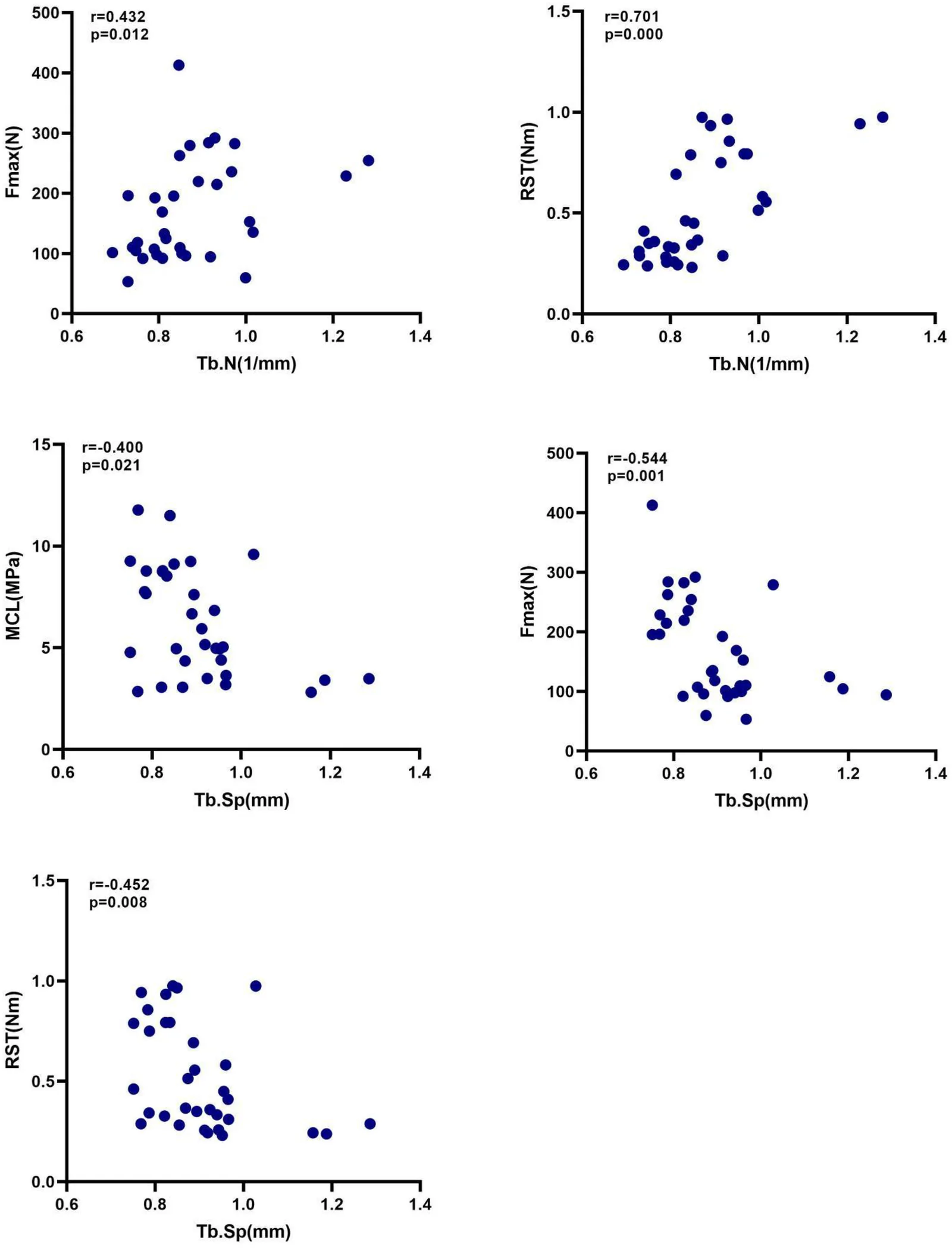
Relationship between microstructural indices and biomechanical parameters. * *p* < 0.05 statistically different, ** *p* < 0.01 statistically different.

## 4 Discussion

This study found that the parameters of trabecular microstructure, such as Tb.N, Tb.Th, and BV/TV, are positively correlated with the MCL, RST, and Fmax of the femoral head’s cancellous bone; conversely, Tb.Sp and SMI are negatively correlated with the MCL, RST, and Fmax of the femoral head’s cancellous bone, indicating that changes in bone structure significantly affect bone strength. Variations in the microstructural space and mechanical properties of bone influence bone strength, leading to an overall reduction in bone mass (7).The reduction in trabecular thickness is associated with decreased connectivity of trabecular bone (8). The changes in microstructure lead to a decrease in bone biological quality, reducing the mechanical properties of the bone, increasing brittleness, and raising the risk of fractures; fractures will occur when osteoporosis reaches a certain level (9).

Bone tissue is a type of biological material with a highly complex hierarchical structure, exhibiting different physical and mechanical properties in various directions. The mechanical properties of bone tissue and the structure of trabecular bone are the two main factors determining the mechanical properties of trabecular bone. Fragile fractures that occur in the context of metabolic bone diseases, such as osteoporosis, typically happen in the trabecular regions (10). During the occurrence and development of osteoporotic fractures, the microstructure of bone tissue changes with the reduction of bone mass. In 2001, when the NIH in the United States redefined osteoporosis (11), it particularly emphasized the significance of the mechanical strength of bones in patients with osteoporosis. This fully reflects the important research significance of bone quality in the occurrence of fractures. The intrinsic properties of bone structure and tissue itself determine the mechanical strength of bones (12). The bone density measured by the macroscopic method cannot accurately reflect the impact of changes in the microstructural and material characteristics of bone on bone strength, and these early changes in bone tissue are crucial factors that transition from quantitative to qualitative changes, ultimately determining bone strength and the likelihood of fractures (13, 14). The use of bone density as the sole indicator cannot reflect the impact of the subtle geometric features and the heterogeneous density structure on bone strength, nor can it fully explain the occurrence of fractures. The correlation between bone density and bone strength is not based on mechanical principles, and attention should be paid to bone quality in the assessment of fracture risk (15, 16).

The research conducted by Chen et al (17) on the microstructure of the tibial trabeculae in ovariectomized rats is similar to our results, indicating that the destruction of the microstructure of trabeculae leads to a reduction in biomechanical performance. The pathological changes of osteoporosis are manifested by the thinning of trabeculae, a decrease in their number, and a reduction in the connection points between trabeculae, resulting in increased load on the remaining trabeculae, which reduces their strength. The geometric shape of trabeculae determines their structural mechanical characteristics, while the microstructure of bone directly affects the material mechanical properties of bone. Changes in bone structure can significantly decrease the strength of the bone. Yamada S et al. (18) studied that patients with osteoporotic litigation exhibit a significant thinning of trabecular bone, with some trabeculae interrupted and gradually absorbed, an increased distance between trabeculae, and damage to trabecular connections, which can lead to a decrease in the overall mechanical strength of cancellous bone.

The BV/TV is the percentage of trabecular volume relative to the total bone tissue volume, which is a key microstructural indicator determining the overall mechanical properties of trabecular bone (5). The research by Nazarian et al. (20)also indicates that fractures of the trabecular bone in human vertebrae are more likely to occur in areas with low BV/TV, where BV/TV is the best microstructural factor for predicting fractures. SMI represents the ratio of plate-like structures to rod-like structures; theoretically, the ideal value for a complete rod-like structure is 3, while the ideal value for a plate-like structure is 0.The research (20) found that with the increase in age, the SMI of trabecular bone gradually increases. In the animal model of ovarian removal, 17 SMI is negatively correlated with bone density, meaning that as the bone density decreases due to osteoporosis, SMI increases, and the trabecular structure approaches a rod-like structure. The research conducted by Poilliot et al. (21) indicates that BV/TV and SMI are significant predictive factors related to Young’s modulus, reflecting the mechanical properties of bone. Our study found that the results of the biomechanical experiments in the laboratory were significantly positively correlated with BV/TV and significantly negatively correlated with SMI. This suggests that BV/TV and SMI are important related factors in the biomechanical properties of osteoporotic femoral heads. The research by GongH et al. (22) found that the vertebral high SMI region is more prone to fractures and further demonstrated that the bone strength in this region is decreased.

The femoral neck fracture is the result of various stresses (compression, shear, bending, torsion) acting at anatomically weak points. In the study of the relationship between bone microstructure and biomechanical experiments (23), most research has primarily been based on three-dimensional finite element analysis, while there is relatively little research assessing trabecular strength through mechanical testing of its ultimate load. This study evaluates the relationship between changes in bone microstructure and fracture quality by simulating injury mechanisms such as compression, shear, bending, and torsion. Although this research provides an in-depth analysis of the relationship between the microstructure of trabecular bone and its biomechanical properties, there are still some limitations that may affect the general applicability and interpretation of the findings. First, prominent baseline age discrepancy exists between experimental group (80.65 ± 8.85 years) and control group (64.08 ± 6.93 years). Age is a well-known strong confounding factor altering trabecular microarchitecture and bone biomechanical behaviours. We did not implement age-matching for specimen enrolment and cannot perform statistical adjustment for age based on current dataset. Hence, inter-group differences in bone microstructure and biomechanical indices may be partially driven by age rather than osteoporosis alone. Second, control specimens were harvested from patients with hip degenerative disease rather than healthy subjects. Hip osteoarthritis may trigger osteosclerosis and trabecular bone remodelling, which potentially confound Micro-CT microstructure measurement and biomechanical testing outcomes. Third, the sample size of this retrospective ex-vivo specimen study is limited (*n* = 20 for experimental group, *n* = 13 for control group). Prospective statistical power analysis was not performed before sample collection; observed correlation coefficients are exploratory and may carry overfitting risk. Our correlation findings require further independent larger-sample validation. Fourth, all bone specimens were preserved in 10% neutral buffered formalin. Formalin fixation may alter bone mechanical properties compared with fresh-frozen bone; absolute values of biomechanical parameters in our study may deviate from fresh bone, though relative group-wise comparisons remain valid given unified fixation protocols for all samples. Fifth, despite standardised sampling location, orientation and operator procedures, residual sampling variability may still introduce experimental noise. Sixth, partial-volume effect and threshold-setting bias cannot be fully excluded under current 36 μm Micro-CT voxel resolution when quantifying trabecular thickness. Finally, all experimental data were obtained from ex-vivo isolated femoral-head specimens under laboratory conditions. Correlations observed in bone samples cannot be directly extrapolated to predict clinical fracture risk for living individual patients. In addition, the parameters and equipment used in biomechanical testing may affect the consistency and comparability of the results, and the limitations of the experimental design may lead to insufficient reproducibility of the results. Finally, the singularity of the research methods is also a significant limitation. Although this study integrates the analysis of microstructure and biomechanical performance, it fails to conduct a comprehensive assessment and analysis of other factors influencing osteoporosis and fracture risk, such as nutritional status, medication treatment, and exercise levels. This may result in an incomplete understanding of the risk factors for osteoporotic fractures, thereby affecting the reliability of the research conclusions.

In terms of future research directions, consideration can be given to investigating the dynamic changes in the microstructure of trabecular bone from a longitudinal study perspective, with a longer follow-up period, and its relationship with fracture risk. Furthermore, researchers may explore the effects of different intervention measures on the microstructure of trabecular bone and its biomechanical properties to determine which treatment plans can effectively improve patients’ bone quality and reduce fracture risk. Conducting multicenter collaborative studies is also a direction worth considering, as it would allow for the collection of larger sample data, thereby enhancing the generalizability and reliability of the research findings. For the prevention and treatment of osteoporosis, deeper interdisciplinary collaboration should be encouraged, integrating advancements in bone biology, imaging, and biomechanics to promote evidence-based clinical applications. At the same time, society should strengthen health education regarding osteoporosis and fracture risk, raising public awareness and importance of this disease to promote early intervention and the establishment of healthy lifestyles, ultimately improving the quality of life and health status of the elderly population. Through these efforts, it is hoped that more effective osteoporosis management and fracture prevention can be achieved in the future, truly reducing the burden of this disease on society and families.

## 5 Conclusion

This ex-vivo specimen-based study shows that compared with the control group, patients with osteoporotic fractures exhibit significant destruction of trabecular microstructure and a marked reduction in biomechanical properties. The destruction of trabecular microstructure is significantly correlated with the decrease in its biomechanical properties. These results only reflect bone-quality characteristics of isolated femoral-head cancellous bone specimens under laboratory conditions. Considering confounding factors including baseline age imbalance and potential bone remodelling among control subjects, findings should not be directly extrapolated to evaluate or predict clinical fracture risk of living patients. Nevertheless, these results are derived from limited-size ex-vivo specimens with several confounding factors as mentioned above and should not be directly used for individual clinical fracture-risk prediction. Therefore, the changes in the trabecular microstructure and biomechanical properties of the femoral head cancellous bone *in vitro* can intuitively reflect the changes in bone quality of the patients, providing a possible theoretical basis for seeking better treatment measures.

## Data Availability

The original contributions presented in the study are included in the article/Supplementary Material, further inquiries can be directed to the corresponding author.
